# Family involvement in nursing homes: an interpretative synthesis of literature

**DOI:** 10.1177/09697330221085774

**Published:** 2022-06-22

**Authors:** Nina Hovenga, Elleke Landeweer, Sytse Zuidema, Carlo Leget

**Affiliations:** 10173 University Medical Center Groningen, Netherlands; University of Humanistic Studies, Netherlands

**Keywords:** Family involvement, family–staff relationship, nursing home, issues, trust, care ethical

## Abstract

**Background:**

Family involvement in nursing homes is generally recognized as highly valuable for residents, staff and family members. However, family involvement continues to be challenging in practice.

**Aim:**

To contribute to the dialogue about family involvement and develop strategies to improve family involvement in the nursing home.

**Methods:**

This interpretative synthesis consists of a thematic analysis and care ethical interpretation of issues regarding family involvement from the perspective of families in nursing homes reported in literature.

**Findings:**

This study reveals the complexities of family involvement in the nursing home by drawing attention to the moral dimension of the issues experienced by families, as seen through the theoretical lens of Baier’s care ethical concept of trust as a theoretical lens. The synthesis of literature resulted in a thematic categorization of issues reported by families, namely, family–staff relationship, psychosocial factors and organizational circumstances. The care ethical interpretation of the synthesis of literature showed that the concept of trust resonates with all reported issues. Trust evolves over time. Early issues are mostly related to getting to know each other. Secondly, families want to experience that staff are competent and of good will. Difficult feelings families may have, such as guilt or loneliness, and dealing with the deterioration of the loved one puts families in a vulnerable position. This power imbalance between family and staff impedes a trusting relationship. Issues related to organizational circumstances, such as understaffing, also undermine families' trust in staff and the nursing home.

**Discussion and conclusion:**

Baier’s theoretical concept of trust provides a deeper insight into the moral dimension of family involvement from the perspective of families in the nursing home. To improve family involvement in practice, we propose to aim future interventions at reinforcing trust in the relationship between family and staff as well as in the organizational context in which these care relationships occur.

## Introduction

In the past decades, research has demonstrated that family members and other loved ones often stay involved in the care for their relatives after placement in a nursing home.^1–3^ Family involvement in nursing homes is expressed in various ways. It includes instrumental aspects of care such as personal care and daily living assistance, taking the loved one to outings and taking care of financial affairs.^4^ In addition, families often provide non-instrumental care, for example, visiting, socio-emotional support, monitoring provision of care and representing the interests of their relatives in the nursing home.^2,5,6^ In this study, we opt for a broad definition of family involvement including both instrumental and non-instrumental care activities aimed at the well-being of the resident. Family involvement in care for residents living in nursing homes is generally recognized as highly valuable. For example, family members can provide insight into the resident’s personal history and preferences and so help staff to deliver personalized and relational care.^6–8^ This positively affects quality of life and well-being of both residents and their families.^8–14^ Because families often know their loved one well, cooperation between families and staff in dealing with, for example, aggressive behaviour, can be very helpful.^15^ In addition, family involvement can help both residents and family members to cope with emotions related to moving to a nursing home.^16^ Staff–family collaboration in the care for residents can contribute to a better work environment in the nursing home. It can prevent or reduce emotional tensions and conflicts between families and staff,^17^ prevent burnout among staff members and reduce high staff turnover.^18^

However, despite these advantages, family involvement continues to be challenging for both staff and families. Staff, for example, may have issues with families being too demanding and/or expecting immediate action. Often this springs from misunderstandings about what families can expect from staff in the daily practice of the nursing homes.^19^ Families report struggling with issues related to power, communication,^20^ role differences and dependencies.^21^

Previous research on family involvement revealed a multitude of issues. Finding an underlying connection between those issues, a common value, could help us gain a deeper understanding of the problem and formulate recommendations for practice. As care ethics starts from what is at stake for people in practice,^22^ care ethical interpretation may help us to discover this common value for family involvement. Moreover, care ethics focuses specifically on addressing issues related to relationality and places them in the organizational and political context,^22^ aspects which in literature are recognized as highly important to overcome barriers to family involvement in the nursing home.^6,20–24^ A synthesis of literature and interpretation of the findings will contribute to the dialogue about and to develop improvement strategies for family involvement in nursing homes.

Our literature synthesis question in this study is: ‘What are the issues of family involvement in nursing homes reported by families?’ (Part I). After that, we address the following interpretative question: ‘Is any coherence observable between these issues from a care ethical perspective?’ (Part II). Finally, we will discuss the usefulness of the findings of Part II to formulate recommendations to improve family involvement in practice.

## Methods

Interpretative synthesis of literature was chosen for this inquiry. Unlike integrative synthesis, this methodology aims to develop theory rather than summarize data.^25^ The results of interpretative synthesis mainly depend on the diversity of concepts found in the literature.^26^ Interpretative synthesis is appropriate here because it allowed us to search for issues in the empirical studies and subsequently reflect on them. This enabled us to develop further insights into what is morally at stake in family involvement in nursing homes from the perspective of family members. Our methodological approach therefore consists of two parts. First, we describe the method used to map the literature on the issues experienced by family members in the context of family involvement in the nursing home. Then we describe how a care ethical perspective resulted in a more comprehensive understanding of these issues.

### Search strategy

We searched Medline/Pubmed, PsychInfo, Cinahl and Web of Science. Subject areas used to identify literature were as follows: (1) nursing homes; (2) family; (3) involvement and (4) issues. Synonyms were identified for each component to enhance completeness. With this search strategy, we aimed to find relevant studies on the broad range of issues and challenges experienced by family members in their involvement in the care of their loved ones who live in a nursing home. Appendix 1 Table 2 provides the detailed description of the definitive version of the search strategy. With the use of those terms, we searched for references defined in subject heading or title/abstract. The final search was conducted on 25 November 2019.

### Inclusion/exclusion criteria

Inclusion criteria for the interpretative synthesis were as follows: (1) the article contains empirical data about issues experienced by families in the context of family involvement; (2) the setting provides 24-h nursing services, for example, referred to as a nursing home or long-term care facility; (3) the target group are family members of residents, family member was broadly defined, including next-of-kin as well as significant others; (4) the study is published in an English language peer-reviewed journal and (5) publication date between 2000 and 2019. We excluded opinion articles, conceptual papers and reviews. We checked the references in the reviews for relevant studies that included empirical data about issues experienced by families regarding family involvement in a setting where 24-h nursing services are provided. We did not find relevant additional studies.

### Selection process

**Figure 1.**
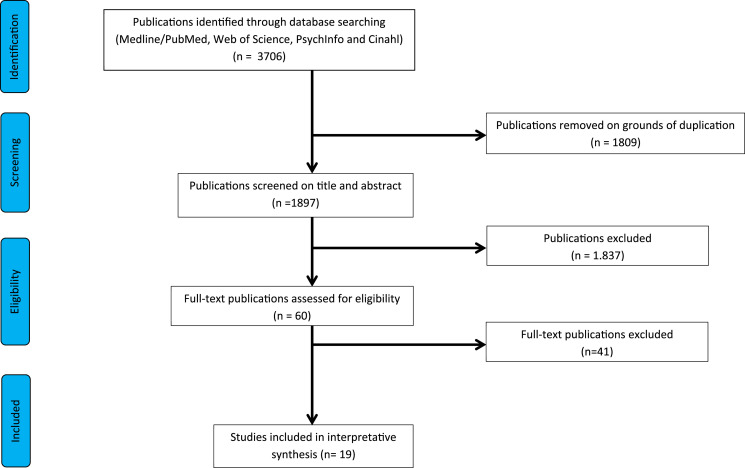
PRISMA flow chart of the study selection.

The initial search yielded 3706 search results. All were imported into a reference management tool (Endnote) for duplicate screening and removal, which resulted in a total of 1809 articles. Based on the inclusion and exclusion criteria, the first two authors independently screened all titles and abstracts for relevance to the research question. If in doubt, the publication was included for further evaluation. This resulted in a selection of 60 potentially relevant articles. Subsequently, a full text review of remaining papers was conducted by the first author. The second author read and evaluated half of the full text articles, and the articles about whose inclusion or exclusion the first author was not sure. Ultimately, 19 articles were included in this interpretative synthesis (Figure 1).

### Data extraction

The studies included were read and re-read by the first two authors to gain familiarity with the data. During this process, the first two authors focussed on issues experienced by families related to family involvement described in the result sections of the included articles. It became apparent that many of the study findings not only described issues but also factors that reduced or prevented issues and/or were experienced by family members as beneficial to family involvement. In order not to miss relevant data, we decided to collect these facilitating factors as well. Next, data from each study about aim, year of publication, study design, participants and country were extracted. All data were extracted into a spreadsheet.

### Data analysis part I: Synthesis of issues

Thematic analysis was used as a method to synthesize the issues and facilitating factors extracted from the included studies. This flexible method allows identification of prominent and recurrent themes in the extracted data.^25^ The thematic analysis consisted of two steps: coding extracted data and developing descriptive (sub)themes.^26^ In step one, the first two authors identified the underlying issues reflected by the facilitating factors. They then independently coded each issue inductively, staying close to the themes as identified in the literature itself. However, the themes could not simply be translated into codes. When appropriate, new codes were developed and checked for consistency of interpretation. In step two, the first two authors looked for similarities and differences between the codes in order to group them in a tree structure. This iterative process included several discussions between all four authors about how to cluster the issues, that is, which issues related to which (sub)themes.

### Data analysis part II: Care ethical interpretation of the synthesis of issues

For the secondary analysis, the authors used a care ethical theoretical framework to reflect on the results of part 1 (synthesis of issues). The methodology of care ethics is a form of empirical ethics research.^22,27^ It focuses on fostering a dialogue between empirical and conceptual research, enabling the researchers to draw normative conclusions. The authors first looked for an overarching theme that resonated with all reported issues and could help to understand any underlying value(s). The four authors then jointly discussed which theoretical framework would best help to understand the underlying connectedness of the results of the first part of the analysis (synthesis of issues).

## Results

### Results Part I: Synthesis of issues

Nineteen articles (Figure 1) from six different countries were included in the interpretative synthesis. The majority of the studies have been conducted in Canada,^15,28–31^ followed by Sweden^7,10,32–34^ and Australia.^8,9,13,35^ Other articles originated from the United States,^11,12,18^ the United Kingdom^14^ and Norway.^36^ The studies used various research strategies: a qualitative approach employing an interpretivist design,^13,15^ grounded theory,^7,14,30,32^ content analysis,^9,10,33^ ethnography,^31,34^ phenomenology-hemeneutics,^36^ participatory action research,^28^ descriptive,^11^ thematic and a conversational approach^29^ as well as a case study approach.^12^ Two studies used mixed methods (survey-based/grounded theory)^18,35^ and one study used a quantitative design (survey-based).^8^ Thirteen studies only included family involvement issues addressed from the perspective of family members.^8–12,14,15,29,30,32–34,36^ Six studies described the experiences from more than one perspective, of which four involved family members and staff^7, 13, 18, 28^ and two included staff members as well as family members and/or residents.^31, 35^ Categorization of the collected issues addressed from the perspective of family members resulted in three main themes with subthemes and codes. See Table 1 for an overview.

**Table 1.** Thematic synthesis of issues in family involvement in the nursing home from the family perspective.

Theme	Subtheme	Code
Family–staff relationship	Not-knowing-each-other	Family members not knowing staff and nursing home
		Family members and resident not known by staff
	Redefining the caregiving role	Difficulties relinquishing/holding on to previous role
		Difficulties re-establishing the familial relationship
	Care expectations	Disappointment in care delivery of staff
		Disappointment in competence of staff
	Communication	Not being informed
		Giving and receiving criticism
		Not being taken seriously
Psychosocial factors	Feelings about a relative’s move to nursing home	Guilt
		Loneliness
		Other feelings
	Dealing with the deterioration of the relative	Difficulty to connect with the relative
		Difficult to accept changes in the relationship with the relative
	Personal circumstances	Competing demands
		Distance
Organizational circumstances	Staffing	Understaffing
		High staff turnover
	Atmosphere	Unfriendly staff
		Untidyand unhygienic surroundings

### Family–staff relationship

#### Not-knowing-each-other

Several studies report that, for family involvement, it is important for families that they not only get to know the staff and the nursing home^28,29,35^ but also that they and their loved ones are known by the staff.^11,12,28,33,35,36^ Lack of initiative on the part of the staff to get to know each other, as well as finding the door closed every time you try to talk to staff, is reported as obstacles to building a relationship with staff, especially when the resident has just moved to the nursing home.^29^ Families indicate that not being introduced to the most important people involved in the care for their loved one and not knowing who is responsible for what impedes getting to know each other.^28,35^ In addition, families report the need for information about the organization^29^ and being shown around the entire nursing home.^35^ Not meeting these needs prevents family members from getting to know the organizational context.

Families find it annoying if staff do not know or welcome them by name.^28,35,36^ It may make them feel that if staff are not interested in them, they might also not be concerned about their loved one.^28^ Families also report being disappointed if staff does not pay attention to what they tell them about the background, needs and wishes of their loved ones.^33,35^

#### Redefining the caregiving role

A relative moving to a nursing home often marks the end of one type of caregiving and the beginning of a new caregiving role for families.^29^ This can bring multiple challenges. Some families report finding it difficult to relinquish their previous caregiving role to the nursing home staff^11,12,18,29^because, for example, they feel they are losing control.^18,29^ ‘Not caring for him oneself, giving him to strangers’.^18^ Families who wish to continue their previous caregiving role as much as possible^14,29,35^ may experience negotiating changes in roles with staff members over time as difficult.^14^ ‘I sometimes find it difficult to know where the lines of responsibility stop and start when it comes to the home and to the family’.^14^

Families report finding it challenging to change their hands-on caregiving role into a more ‘familial relationship’^11,12,29^ which means just being together and giving emotional support.^33,34^ This new caregiving role require skills other than the instrumental skills of assisting in personal care of daily living, which some families struggle with.^29^

#### Care expectations

Families may have expectations regarding staff that are not always in line with the actual care provided. They are not always confident that their loved one receives good care.^11,34^ Examples mentioned are that their loved one’s daily needs are not met,^15,33,36^ safe care is not provided,^11^ the care provision lacks dignity^33^ and/or the loved one’s wishes are not respected.^33^ Families also experience it as problematic when staff fail to make an effort to bring out the loved one’s personality, sociability and sense of humour and thereby maintain their identity^11,12^ Especially ‘little things’ can be a source of stress and frustration for family members.^14,28,33,36^ ‘Little things’ that are mentioned include details regarding personal appearance (e.g. stains on dress), clothes going missing, food preferences not being respected^14,28,33,36^ and/or nursing staff not showing enough interest in their loved ones.^36^ In addition, families report having concerns about their loved one’s psychosocial well-being.^10^ They do not want them to be bored or lonely for long periods of time^7,36^ and therefore often advocate the necessity of sufficient recreational activities and interaction with other residents and staff.^7,11,35^ Another concern is staff not taking initiatives to develop necessary improvements in care, especially after families having insisted on improving care and not receiving any response.^7,10,12,33^ Families also stress that staff members do not always share important information about their loved ones with their colleagues.^7^

Families furthermore report doubts about the staff’s care competencies,^12,15^ mentioning a lack of medical competency,^36^ disinterested physicians and inexperienced staff who lack knowledge about how to care for their relative.^33^ Staff’s lack of care competencies causes distrust in families,^9,36^ which in turn can lead to feelings of insecurity and concern.^7^ When care expectations are not met, families feel they have to continuously observe staff–resident interactions to monitor the care and the well-being of their loved one.^9, 30^

#### Communication

Families report the information about their loved one’s well-being and about the provided care is sometimes insufficient,^7,13,30,33,35^ inadequate,^7,18^ unreliable^34^ and/or hard to obtain.^7,33,35^ Several families report multiple obstacles to making contact with staff, such as staff keeping a distance and in many cases being absent.^10^ In addition, families say they miss informal moments during their visits to ask questions^33,35,36^ and to chat and discuss how their relative is doing.^7,35^ When they do talk to staff members, families report they are not always able to answer their questions or resolve their concerns.^13,28^ Families also feel frustrated because they generally feel that staff just want to convey information and do not want to start a real conversation.^13^ Also, not receiving essential information concerning their loved one spontaneously from staff is considered a problem. Families want to be informed without having to ask.^7,13,35^ They report feeling that the responsibility for interaction and communication with staff is all on them.^10,13^ ‘Staff should tell us about resident progress before we have to ask’.^35^

Several families state they want to communicate their concerns to staff members^11^ but are reluctant to do so as their concerns could be construed as criticism. This might offend staff and could backfire on them or their loved one.^7^ Families do not want to be a nuisance, or feel like they are nagging or complaining.^14,28,35^ According to some families, staff are not open to feedback.^18^ Vice versa, some families also find it difficult to be criticized by staff, for example, for not being involved enough in the care.^18^

Families report they want to be taken seriously, for example, be asked to participate and invited as active participants in care planning and reviews.^7,10,13,35^ According to families, an inclusive environment requires openness.^36^ They mention sometimes feeling like outsiders because they have too little influence on the care of their loved one.^32^ They feel this is because staff do not always take into account their knowledge and wishes regarding the care for their loved one^10,32^ and sometimes misunderstand the extent to which families want to be involved in decision-making.^9,36^ Families indicate that they would like to be invited to care conferences more often.^31,33^ However, when they are invited to these meetings, their concerns often remain unaddressed or their knowledge is treated as subordinate to expert knowledge.^31^

### Psychosocial factors

#### Feelings

The admission of a loved one in a nursing home is often accompanied by mixed and ambivalent feelings for families. On the one hand, family members may experience positive feelings, like a sense of freedom and relief. A sense of freedom because they can relinquish certain hands-on aspects of care,^32^ and relief knowing that their loved one is in a safe and structured environment where they will receive the care they need.^11^ But they also report feelings of guilt, for example, about the placement decision,^15,18,32^ not doing enough in terms of caring for their loved one in the nursing home,^36^ not being with their relative as much as they should or feeling healthy while their loved one’s health deteriorates.^33^

Family members also struggle with the loneliness that comes with various experiences of loss, such as loss of friends and a supportive network^29^ and loss of family structure and home (no longer living together).^29,32^

In addition, families report feelings of anxiety when visiting their loved one^29^ and fear about their own ageing and dying resulting from the confrontation with their relative’s deterioration.^34^ Feelings of exhaustion after a long period of caring at home are also reported.^32^ One study associates feelings of powerlessness with not having a real choice about which home to place their relative in.^32^ Families often experience a lack of recognition of their feelings by staff, especially during the end-of-life phase.^33^

#### Dealing with the deterioration of the relative

Several studies report that families experience a sense of sadness watching the gradual decline of a loved one.^8,15,33^ Due to cognitive decline, their relative may no longer be the person he or she once was and family members face the challenge of acknowledging this loss of identity.^11,34^ Deterioration of their relative may also change their relationship.^33,34^ Reduced responsiveness of their loved one may make it difficult for families to connect or sustain a conversation with the resident^15,33^ and families sometimes report no longer knowing what to do during a visit.^35^ As a result of the difficulty to connect, they sometimes limit the frequency of visits.^15^ ‘The hardest thing about visiting Mum is that you just cannot keep up a conversation’.^35^ Some families also have difficulty understanding their relative’s diagnosis and its consequences.^11,13^

#### Personal circumstances

Other responsibilities and competing demands in life, such as full-time employment^8,15,33^ or family commitments,^8^ are reported as reasons to visit less often. Families living far away from the nursing home^8,33^ and having inadequate access to transportation also affects visiting opportunities.^8^

### Organizational circumstances

#### Staffing

Frequent rotation of staff, understaffing and agency staff are reported to impede getting to know each other, which directly impacts the family–staff relationship.^28^ Families experience distrust in care delivery when there are problems of continuity of staffing^9^ or understaffing.^14^ Understaffing is also experienced as a barrier to good communication, and high staff turnover sometimes causes a sense of exhaustion.^18^ Families lack the energy to build another relationship with new staff.^7^

#### Atmosphere

Some studies report that families are not always comfortable with the atmosphere of a facility. This can affect open communication and a more collaborative relationship with the staff.^9,36^ Descriptions of an unpleasant atmosphere include staff being unfriendly and difficult to approach.^9^ Secondary to the dynamics of interaction are the physical aspects of the facility, for example, an unpleasant smell and the environment being untidy and/or unhygienic .^9,35,36^ ‘There is no ‘smell’ of a kind you often get in these places. It is nice the way it smells of cooking here. I think that means a lot to the old people here’.^26,36^

## Results part II: Care ethical interpretation of the synthesis of issues

During the interpretative care ethical analysis conducted within the research group, the concept of trust emerged as the most central underlying common value to better understand the relationship between the issues, for three reasons. First, families literally refer to (dis)trust several times in their descriptions of experienced issues. For example, they indicate that if staff are incompetent,^9,36^ their care expectations are not met^11,34^ or how discontinuity of staffing^9^ leads to mistrust. Second, most of the issues reported by families were directly related to the relationship between family members and staff. This implies that the relational dimension in family involvement is important. Earlier literature points to the importance of trust in realizing beneficial family involvement.^20,37^ Finally, our interpretative process builds on a care ethical body of literature that also points to trust as an important aspect of the dynamics of interpersonal relationships.^38^

The result of our literature search for ‘agenda-setting’ authors who had elaborated trust into a care ethical framework led us to the work of American philosopher Annette Baier.^38^ According to Baier’s trust approach, human beings are so interconnected that we need each other to sustain and maintain our lives. As a result, in some instances ‘we have no choice but to allow some others to be in a position to harm us’.^39^ This interdependency of human life is highly relevant in the context of a loved one moving into a nursing home, because specialist care is needed that families are not able to provide. So in line with Baier’s trust approach, families have no choice, but to rely on the competence and good will of staff in the nursing home.^40^ She also characterizes trust as a process that evolves over time and must be placed in the social context.^40^ In summary, according to Baier, trust always involves features such as relationality, vulnerability, power differences, responsibility, dependency and contextuality. These features are also central to the theoretical framework of care ethics,^22^ making Baier’s concept of trust a natural choice for the care ethical interpretation of the synthesis of the issues. In the next paragraph, we describe this interpretation based on the thematic categorization of the issues.

### Family–staff relationship

#### Not-knowing-each-other

Baier considers trust as a process that ‘grows slowly and imperceptibly’^39^ and is a ‘fragile plant’.^40^ In the beginning, trust requires the willingness to give the one we must trust the benefit of the doubt and defer judgement for a while. In other words, wait and see how the trusted person uses his/her discretionary power.^39^ This means that the one we trust is given the freedom to judge what should be done to take good care in a particular situation and act on that.^39^ In the initial period after nursing home admission, families are faced with the challenge of placing the care of their loved one ‘in the hands of’ care professionals, or, to trust that the staff will take good care of their loved one. Especially in the beginning the trust relationship is likely to be fragile because family members and staff do not know each other very well yet. Considering the issues described under the subtheme not-knowing-each-other, this can obviously generate feelings of insecurity in families. According to Baier, trust develops through awareness of the risks we are taking by trusting the other. And by judging whether taking those risks is worth it, or in other words: ‘sustained trust is experienced trust’.^39^ In order to assess whether the risks of trusting staff are acceptable, families at least need to get to know them. Obstacles to getting to know staff, like not meeting all relevant persons and not knowing who is responsible for what,^28,35^ make it difficult for families to build a trusting relationship with staff and reduce their initial insecurity. Conversely, it is also important to families that staff get to know their loved one well,^11,12,28,33,35,36^ so they can trust staff to know how to use their discretionary power appropriately. Disrespecting the wishes of the loved one^33,35^ or not being recognized by staff^28,35,36^ are therefore issues that could jeopardize their trust.

Families also report finding it annoying if they are not recognized by staff.^28,35,36^ According to Baier, trust is to some degree mutual because ‘the risks are on both sides’.^39^ The one who is trusted runs the risk of abusing her/his discretionary power by not doing the right thing and being ‘punished’ for it.^39^ So, a lack of reciprocity in the relationship can increase the risk of staff (unintentionally) misusing their discretionary power. If staff do not know a family or the resident, it would obviously make exercising their discretionary power appropriately difficult, which could damage the fragile trust relationship between families and staff.

Difficulties in getting to know the organizational context^29,35^ can also undermine building a trusting relationship with staff, if we assume, like Baier, that the social context influences trust in individual staff members. Baier not only sees trust as something that occurs between two people but places it in a broader, social context by talking about ‘networks of trust’,^40^ ‘climates of trust’^40^ and ‘network of relationships’.^40^ She states that a healthy climate of impersonal trust contributes to the likelihood of strong personal trust relationships and vice versa.^39^

#### Redefining the caregiving role

Baier argues that depending on another’s good will involves vulnerability of the one who trusts because there is always the possibility of being harmed by the one who is trusted.^40^ So, if a loved one is placed in a nursing home, families run the risk that the loved one and the family members themselves are harmed by staff. This implies insecurity for families, especially initially, when they must give staff the benefit of the doubt. Feeling they have no control, families may have difficulty relinquishing their caregiving role to the nursing home staff.^18,29^ For most families, it is a challenge to relinquish certain care responsibilities and change their previous caregiving role into a more familial relationship which focuses on providing emotional support.^11,12,29,33,34^ To be able to do this, families need to develop a sufficient level of trust that staff will take over their caregiving role correctly, so they can focus on their family relationship.

#### Care expectations

Trust is also characterized by expectations, that is, families rely on the competence and good will of the staff.^40^ For example, families expect staff to pay attention to the ‘little things’, for example, serve food according to the preferences of their loved one or pay attention to details of personal appearance.^14,28,33,36^ If those expectations are not met, this is an indication for families that staff are inattentive and, in line with Baier’s trust approach, families are therefore likely to develop (more) distrust towards staff intentions. Families try to maintain control by continuously assessing whether staff is sufficiently competent to take good care of their loved one.^9,30^In line with Baier, families not only judge staff on what should be considered ill will but also on incompetence. A family doubting the competence of the staff leads to a situation of discomfort.^7,9,12,15,33,36^ For example, a lack of medical competency^36^ or staff neglecting to make necessary improvements in care despite persistent involvement ^7,10,12,33^ can undermine trust. As a result, families may start monitoring the performance of staff continuously.^9,30^ This constant checking in turn leads to even more distrust.^39^

#### Communication

In order to gain insight into and assess the risks involved in sustaining trust,^39^ it seems important that families are well informed about the care provided. Judging by the issues experienced by families, this is not always the case.^7,13,18,30,33–35^ Other issues related to communication reported by families included a lack of initiative on the part of staff^7,10,13,35^ as well as not always being taken seriously by staff.^7,9,10,13,31,32,35,36^ One possible underlying cause is that staff are insufficiently aware of their responsibility to use their discretionary power in the most appropriate way. Reciprocity, which is a precondition for a trusting relationship,^39^ seems to be at stake here. Another issue that seems to undermine trust is that staff does not always pass on the information about the family’s loved one to their colleagues.^7^ In line with Baier, who sees trust as ‘networks or relationships’,^40^ this not only affects the trust relationship between families and staff but also the extent to which families have confidence in the cooperation between staff members. Finally, families reported that they are reluctant to criticize staff as they are worried they might offend them.^7,14,28,35^ According to Baier, this fear of offending the other undermines the trust relationship as we must not be too afraid to offend the other when we check the performance of the one we trust.^39^

### Psychosocial factors

#### Feelings, dealing with the deterioration of the relative and personal circumstances

After placement of a loved one in a nursing home, families have no choice but to trust staff and the nursing home, especially initially. In line with Baier, by this act of trust, families end up in a vulnerable position because staff can harm them by not taking good care of their loved one.^40^ This vulnerable position of families can be exacerbated because families have to deal with issues related to different kinds of personal (ambivalent) feelings,^11,15,18,29,32–34,36^ the deterioration of the condition of their relative^8,11,13,15,33–35^ and personal circumstances.^8,15,33^ According to Baier, the vulnerable position of the one who trusts also alters the balance of power between the one who trust and the trusted.^39^ This inequality of power is inevitable according to Baier,^39^ but she emphasizes the need to reveal these power differences to prevent the potential abuse of power.^39^

### Organizational circumstances

#### Staffing and atmosphere

Families reported issues related to staff.^7,9,14,18,28^ It is likely that issues like understaffing or temporary staff undermine the trust relationship between staff and families. In case of understaffing, for example, even if staff are of good will, they may not be able to do what they think should be done to provide good care in a particular situation. Families also reported issues related to atmosphere.^9,35,36^ Unfriendly staff and an environment that is not tidy and clean can make family feel the nursing home is not a pleasant place to be. In addition, it is likely that families see care for the environment as an indication of the care given to their loved one. These organizational obstacles can negatively influence families’ trust in staff. According to Baier, trust is a social phenomenon in which the environment and other relationships can affect the personal trust relationships.^39^

## Discussion

This interpretative synthesis reveals the complexities of family involvement in the nursing home by drawing attention to the moral dimension of the issues experienced by families, using Baier’s care ethical concept of trust. The synthesis of literature resulted in a thematic categorization of issues reported by families. The first theme, ‘family–staff relationship’, described issues related to family members and staff getting to know each other, redefining the caregiving role, care expectations and communication. The interpretation of these issues using Baier’s trust approach generated the following in-depth insights. It takes time for families to build trust in staff after their loved one is placed in a nursing home. In the beginning, a family’s vulnerability is highest because they are not yet able to estimate the risks of handing over the care. To gain insight into and assess the risks involved in developing trust, families may want to check staff performance. If these ‘checks’ are not performed carefully enough, it may impede building a trusting relationship between a family and staff. We discovered that most of the issues in the family–staff relationship are related to the pursuit of the lived experience of trust.

The second theme, ‘psychosocial factors’, illuminated issues concerning families’ mixed and negative feelings, difficulties dealing with the deterioration of the relative and personal circumstances that make involvement more difficult. The care ethical reflection on these issues revealed that the vulnerability of families can increase and with it the power imbalance in the relationship with staff. This puts families in a position of even greater dependency, which can make it more difficult for them to trust staff. Finally, the third theme, ‘organizational circumstances’, showed issues related to staffing and atmosphere. According to Baier, trust is a social phenomenon, meaning that social and other environmental factors influence trust relationships.^39^ The care ethical reflection on the issues showed us that organizational circumstances such as a shortage of staff and/or an untidy and unhygienic environment undermine trust of families in staff and the nursing home.

### Limitations

One limitation of this interpretative synthesis is that our literature search focussed only on the issues experienced by family members. In this study, we miss the perspectives of other directly involved stakeholders, such as staff and the residents. Second, (almost) all studies mainly included families who have a close relationship with their relative. We therefore have no insight in the perspective of the less involved families. Third, our study included results from six different countries, so it is possible that we missed cultural differences in the family perspective on family involvement. Finally, the literature search was limited to the English language. We therefore may have missed relevant literature published in other languages.

### Recommendations

It is our hypothesis that to improve family involvement in the nursing home, the interventions should be aimed at reinforcing trust within the family–staff relationship and in the whole context of the nursing home. First, it is important that staff as well as family are made (more) aware of the moral dimension of the issues family members and they themselves experience regarding family involvement in the nursing home, and how trust plays a role in this. This awareness can be promoted by providing ethics support, for example, by developing competencies in moral reflection of families and staff and the role of trust. Second, as power differences always play a role in caring relationships, staff and families should be more aware of the power imbalance in their relationship and discuss how to deal with it constructively.^39,41^ We also advise staff to, for example, carefully consider the input of family members in care conferences.^31,33^ It would also be helpful if staff make themselves more vulnerable in the relationship with a family by expressing their own considerations about what is the right thing to do and inviting the families’ thoughts about the topic. Third, to build a trusting relationship with families, staff have to realize that they have a responsibility to use their discretionary power in the most appropriate way. As Baier points out, the consciousness of this moral appeal strengthens reciprocity in the relationship, which in turn enhances trust in the relationship.^39^ In order to judge what should be done to provide good care in a particular situation and act on that,^39^ we recommend a continuous dialogue between staff and family members from the start. This dialogue should initially focus on getting to know each other, subsequently on investigating what is important for whom, and finally on what families and staff expect from each other. After that, regular reflection in an open conversation with each other whether ‘the right thing’ is still being done (‘careful’ checks) and, if necessary, adjusts the mutual expectations. Baier speaks of trust as a process that incorporates an element of reflexivity.^40^ Although families also have a responsibility in this, it is important that staff initiate these dialogues, because families now often feel that it is all up to them.^7,10,13,35^ Fourth, staff could become more sensitive to the feelings of families and give them the social-emotional support they need. In addition, it would make sense if staff help families to cope with the declining condition of their loved one, for example, by regularly discussing it with each other and/or providing general information. These interventions can reduce the vulnerable position of families and therefore enhance trust. Finally, management must ensure the presence of sufficient, competent and permanent staff and invest in a clean and hospitable environment. These context-oriented measures will also increase trust in the relationship between staff and family members. All the above actions, when implemented in regular care, will be helpful to enhance a trusting relationship between staff and family members and so improve family involvement in the nursing home. Implementation research is needed to assess, further develop and evaluate these recommendations. Further research should also be aimed at strengthening our understanding of trust in the relationship between staff and family members.

## Conclusion

This interpretative synthesis of literature reveals that most of the issues family members experience in family involvement in the nursing home are related to the relationship with staff, are influenced by personal aspects of family members and by organizational circumstances. The interpretation of the synthesis of issues from a care ethical perspective reveals that trust is an appropriate moral concept for a better understanding of the underlying coherence between the issues.

Using Baier’s theoretical concept of trust as a lens to interpret the overview of issues gave us insight into what is morally at stake in family involvement in the nursing home from the perspective of families. To improve family involvement in the nursing home, the interventions should therefore be aimed at reinforcing trust in the relationship between staff and family members as well as in the organizational context in which these care relationships occur.

## Appendix 1Table 2. Sample of search terms: Database Medline/Pubmed.

(“Nursing Homes” [Mesh] OR “Homes for the Aged” [Mesh] OR “Long-Term Care” [Mesh] OR longterm care [tiab] OR long-term care [tiab] OR nursing home*[tiab] OR assisted living [tiab] OR “Care homes” [tiab] OR homes for the aged [tiab] OR (resident*[tiab] AND (longterm*[tiab] OR long-term*[tiab] OR institut*[tiab])).

AND

(“Dementia” [Mesh] OR dementia*[tiab] OR alzheimer*[tiab] OR nursing home*[tw]).

AND

(“Professional-Family Relations” [Mesh] OR “Family” [Mesh] OR family [tiab] OR families [tiab] OR relatives*[tiab] OR informal carer*[tiab] OR friend*[tiab] OR spous*[tiab]).

AND

(collaborat*[tiab] OR approach [tiab] OR involv*[tiab] OR inclusion*[tiab] OR includ*[tiab] or participat*[tiab] OR engag*[tiab] OR role*[tiab] OR allianc*[tiab] OR triadic care [tiab] OR family care*[tiab] OR visit*[tiab] OR informal care*[tiab]).

AND

(challenge*[tiab] OR barrier*[tiab] OR difficult*[tiab] or dilemma*[tiab] OR obstacle*[tiab] OR moral [tiab] OR ethic*[tiab] OR issue*[tiab]).
